# The hybrid operation based on microsurgery assisted by intraoperative spinal angiography in patients with spinal dural arteriovenous fistula: a series of 45 cases from multicenter research

**DOI:** 10.1186/s41016-024-00372-5

**Published:** 2024-07-19

**Authors:** Xiaorong Sun, Li Yu, Wenqing Jia, Wei Dai

**Affiliations:** 1 Department of Spinal Cord and Spine Ward of Beijing Tiantan Hospital, Capital Medical University, Beijing, 100070 China; 2 Department of Neurosurgery, Nanjing Drum Tower Hospital, Nanjing University Medical School, Nanjing, 210008 China

**Keywords:** Spinal dural arteriovenous fistula, Hybrid operation, Anxiety and depression, Prognosis

## Abstract

**Background:**

To assess the clinical effects of hybrid surgery, which includes spinal angiography-assisted microsurgery, in the treatment of spinal dural arteriovenous fistulas (SDAVF).

**Methods:**

We retrospectively reviewed 45 patients who underwent hybrid Spinal dural arteriovenous fistula (SDAVF) resection between September 2019 and June 2022. The hybrid surgery involved intraoperative digital subtraction angiography (DSA) of the spinal vessels to determine the source of the blood-supplying artery, location of the fistula and draining vein, indocyanine green fluorescence (ICG)-assisted microsurgical resection of the fistula, and postoperative DSA to verify therapeutic efficacy. The Hamilton Anxiety Scale (HAMA), Hamilton Depression Scale (HAMD), Visual Analog Scale (VAS), Barthel score, modified Rankin Scale (mRS) and modified Aminoff-Logue score (key indicator) were used to assess the clinical effects of SDAVF resection.

**Results:**

A series of 45 patients with SDAVF were successfully treated with hybrid surgery without fistula recurrence. There were no intraoperative complications related to spinal angiography, and none of the patients died. Postoperatively, two patients experienced clinical deterioration of spinal cord function, which manifested as bilateral lower extremity paralysis and bladder sphincter dysfunction. Postoperatively, improvement in mALS scores was observed in 16 cases (35.6%) within 1–2 days, 12 cases (26.7%) at 1 week, and 7 cases (15.6%) at 6 months. No SDAVF recurrence was detected in the spinal MRA examination 6 months after surgery. When compared with preoperative mALS scores, 35 cases (77.8%) showed significant improvement in symptoms, 8 cases (17.8%), remained unchanged, and 2 cases (4.4%) deteriorated. Compared with the preoperative scores, the postoperative mALS score was significantly decreased [postoperative vs. preoperative: 2(1,3) vs. 3(2,4)], HAMD score [(12.2 ± 5.5) vs. (19.6 ± 6.3)], HAMA score [(15.6 ± 5.5) vs. (20.5 ± 6.5)], and VAS score [3(2,5) vs. 5(4,8)]. Conversely, Barthel scoresshowed significant increase [(74.6 ± 8.7) vs. (67.8 ± 9.2)] (*P* < 0.05). However, the mRS scores were lower than preoperatively [1(1,2) vs. 2(1,2.5)], but the difference was not statistically significant (*P* > 0.05). There was a significant increase in “good” neurological outcomes at follow-up compared with preoperative function (62.2% vs. 33.3%) (*P* = 0.023).

**Conclusion:**

Hybrid surgery is a safe and effective treatment for patients with SAVF, which is beneficial for improving anxiety, depression, spinal cord, and neurological function, and relieving pain. However, the treatment of patients with SDAVF is a complex, long-term process requiring further multidisciplinary interventions, including clinical care, psychosocial interventions, and neurorehabilitation.

## Background

Spinal dural arteriovenous fistula (SDAVF) is a relatively rare vascular malformation of the spinal cord, where arteries supplying the spinal dura mater or nerve roots crossing the dura mater at the intervertebral foramina communicate with the draining veins of the spinal cord. It contributes to spinal venous reflux and high pressure in the spinal cord veins, leading to spinal cord degeneration and necrosis [1, 2]. In general, patients with SDAVF often present with limb numbness, pain, and urinary dysfunction, which lack specificity [3]. SDAVF has an insidious onset, complex and variable symptoms, and a high rate of disability, necessitating accurate and timely diagnosis and clinical treatment [4, 5]. According to a previous report, the failure of removal is up to 5% in surgically treated SDAVF cases [6]. In addition, endovascular treatment for SDAVFs has certain disadvantages, such as treatment failure, inability to achieve total occlusion, and recurrence [7]. Therefore, further investigation is required to determine the optimal treatment for SDAVF.

Currently, spinal angiography-assisted hybrid microsurgery has shown promise in treating SDAVF. This technique allows for the accurate preoperative localization and intraoperative verification of supplying arteries and draining veins, timely detection of residuals, and effective resection of SDAVF [6]. However, clinical reports on SDAVF resection using this hybrid surgery are limited [6, 8]. Therefore, further investigation is required to determine the potential value of hybrid surgery for treating SDAVF. In this study, 45 patients with SDAVF who underwent hybrid surgery between September 2019 and June 2022 were retrospectively recruited. In addition, various assessment scales, including those for anxiety, depression, quality of life, pain, and Barthel scores, were utilized to assess the outcomes.

## Methods

### Participant selection

**Fig. 1:**
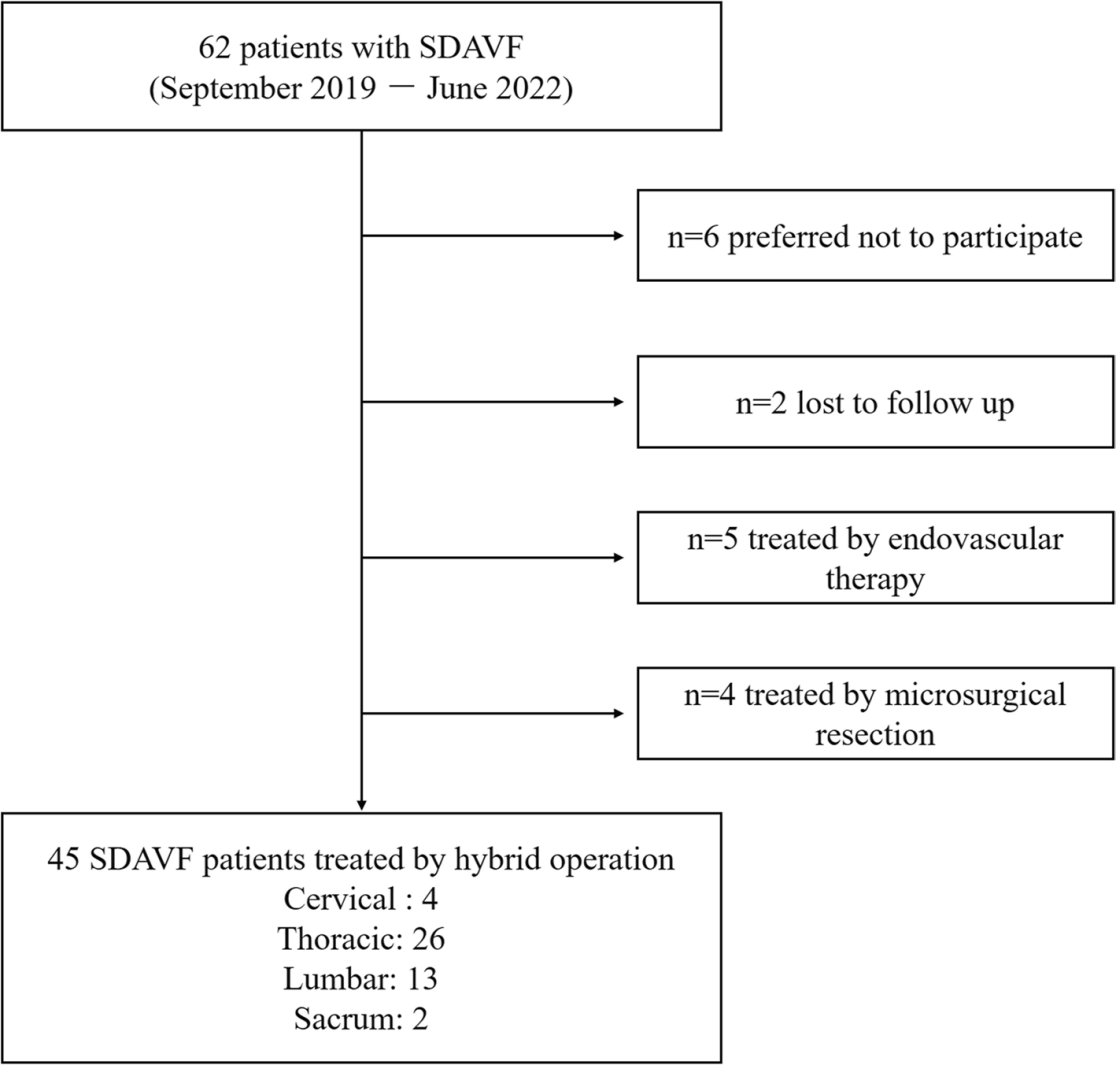
Flowchart of patient selection. SDAVF = Spinal dural arteriovenous fistula

We retrospectively reviewed patients with SDAVF who underwent hybrid surgery between September 2019 and June 2022. The inclusion criteria of this study were as follows: (1) SDAVF suspected through spinal CT angiography or MRI angiography and definitively diagnosed by conventional spinal angiography [9]; (2) SDAVF treated with hybrid surgery involving microsurgery assisted by spinal angiography; (4) patients who volunteered to participate and followed up for at least 6 months after surgery; (5) patients willing to undergo a series of assessments, including the Hamilton Depression (HAMD) scale, Hamilton Anxiety (HAMA) scale, Visual Analogue Scale (VAS), Barthel score, modified Rankin Scale (mRS), and modified Aminoff-Logue score (mALS); (6) patients who underwent magnetic resonance spinal angiography at the 6-month follow-up after surgery. The exclusion criteria were as follows: (1) neurological dysfunction due to spinal disorders (e.g., intervertebral disc herniation and spinal stenosis), (2) comorbid psychiatric or other neurological disorders, (3) interruption of follow-up due to other illnesses, and (4) inability to cooperate in completing the scale test. A flowchart of the SDAVF patient selection is shown in Fig. 1.

This study was approved by the committees of Beijing Tiantan Hospital of Capital Medical University and Nanjing Drum Tower Hospital. Consent was obtained from all patients. Ethical approval numbers were KY-2021–012-02 (Beijing Tiantan Hospital) and Chi CTR200032529 (Nanjing Drum Tower Hospital). Finally, 45 patients diagnosed with SDAVF were enrolled in the study.

### Data collection

The clinical baseline of patients with SDAVF was collected before surgery, including onset age, hypertension, diabetes mellitus, smoking history, time of onset of symptoms to diagnosis, DSA data of spinal cord vessels (including the location of the lesion, number of supplying arteries, and draining veins), HAMD scale, HAMA scale, VAS scores, Barthel scores, mRS score, and modified Aminoff-Logue score profile. The time required for the procedure and the residuals were recorded during surgery. After surgery, changes in clinical symptoms and days of hospitalization were recorded. During the 6-month follow-up, data on HAMD, HAMA, VAS, Barthel, mRS, and mALS were also recorded.

### Surgical procedure

**Fig. 2:**
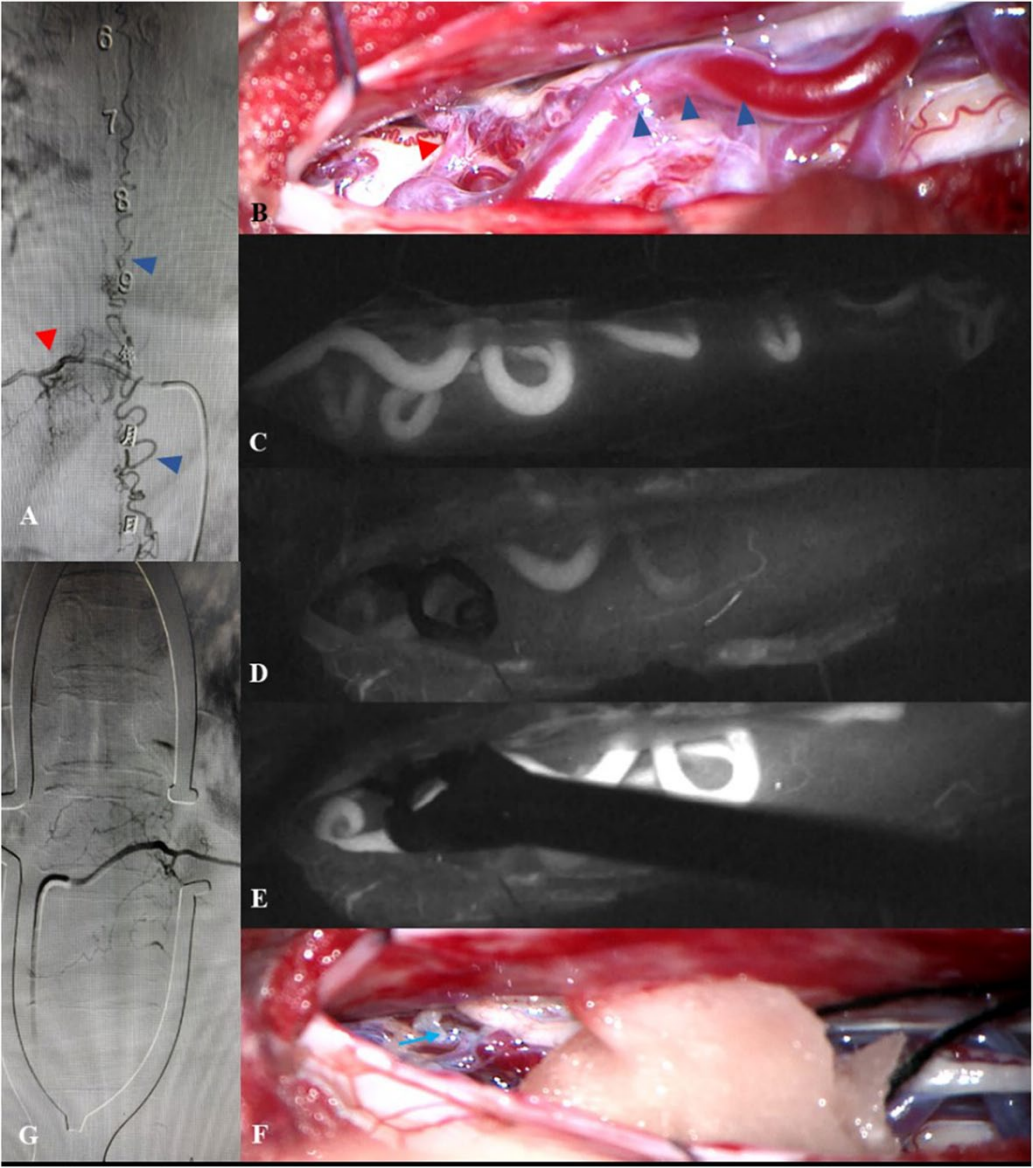
This 57-year-old female patient with bilateral lower limb weakness is initially referred to the orthopedic department with unsatisfactory results and then referred to neurosurgery for spinal myelography, who is diagnosed with SDAVF. The fistula is located at the level of the right thoracic 10 and thoracic 11 (**A**, the red triangle is the responsible artery, and the blue is the draining vein). The responsible artery of SDAVF (the red triangle) and the draining veins are seen to be meandering and dilated during the surgery (the blue triangle) (**B**). The responsible artery and draining veins are confirmed by indocyanine green (ICG) imaging (**C**). Fluoroscopy is performed to confirm the responsible artery and the draining vein (**C**). After the responsible artery is clipped with a temporary aneurysm clip, the draining vein does not appear on fluorescence imaging (**D**). The draining vein reappears after releasing the aneurysm clip (**E**). The responsible artery is electro-coagulated and cut-off (**F**, blue arrows). The SDAVF does not appear on postoperative imaging (**G**, prone position)

During the surgery, evoked neurophysiological monitoring was used to monitor and record the spinal cord function. After administering general anesthesia, the patient was positioned supine, and the right femoral artery was punctured using the modified Seldinger technique with a 5F catheter sheath. A loach guidewire was inserted under fluoroscopic view during the DSA and a 6F long sheath was replaced. The guidewire was removed after the tip reached the common iliac artery, and the angiography catheter was buried in the artery responsible for the SDAVF, in which saline was continuously infused through the pressurized drip. Based on the intraoperative DSA and X-ray fluoroscopy results, the fistula of the SDAVF was located, and the corresponding SDAVF fistula of the upper and lower vertebrae was marked as the incision range. After exposing the paravertebral muscle, grinding drills were used to remove the half-vertebral plate of the SDAVF vertebral body, and a milling cutter was used to open the vertebral plate window. The arteries responsible for the SDAVF, fistula, and drainage veins were identified intraoperatively. After placing a temporary clip on the artery responsible for the SDAVF, indocyanine green (ICG) fluorescence imaging was performed to confirm the absence of the draining vein and to ensure no significant abnormalities in the evoked potentials were observed. The drainage veins reappeared after the temporary clip was removed. After the responsible artery was cut following electrocoagulation, spinal angiography was performed again to confirm the complete removal of the fistula and the absence of any other fistulas (Fig. 2). Finally, the dural matter was closed, and the vertebral plate was fixed.

### Scale evaluation and grading

Patients’ anxiety and depression were evaluated by psychiatrists, mainly using the HAMA to evaluate anxiety and the HAMD to evaluate depression. Scores of less than 7, 7–20, and more than 20 were diagnosed as “none,” “depressive tendency,” and “depression,” respectively [10]. Anxiety was diagnosed using a HAMA scale score of > 14 points [11]. The Visual Analog Scale (VAS) score was used to evaluate pain [12]. The Barthel score was used to evaluate living capacity [13]. The mRS was used to assess neurological outcomes, categorized as follows: 0–1 for good neurological function, 2–3 for moderate to severe disability, and 4–5 for total disability [14]. The Modified Aminoff-Logue Score (mALS) was used as a key indicator to evaluate spinal cord function, including the three dimensions of gait (0–5 points), defecation (0–3 points), and urination (0–3 points). Compared with the preoperative mALS score, a decrease was defined as “improvement,” remaining no change was defined as “no change,” and an increase was defined as “deterioration” [15].

### Statistics

Categorical variables were expressed as *n* (%), and the chi-square test was used. For continuous variables, the data was expressed as mean ± standard deviation (*x* ± *s*), and a *t*-test was used. Data with a skewed distribution were expressed as median (quartile), and the Mann–Whitney *U* test was used. Statistical analyses were performed using SPSS version 25.0 (SPSS Inc.). Statistical significance was set at *P* < 0.05.

## Results

### Patient characteristics

Detailed information is provided in Table 1. A total of 30 male cases and 15 female cases were included in this study, with ages ranging from 17 to 75 years (mean 52.6 ± 14.3 years). Fourteen patients with hypertension, 8 with diabetes mellitus, and 13 with a history of smoking were enrolled. There were 26, 13, 2, and 4 patients in the thoracic, lumbar, sacral, and cervical segments, respectively. Onset symptoms included gait abnormalities in 41 patients (91.1%), abnormal sensation in the lower limbs in 31 patients (68.9%), urinary disorders of varying degrees in 27 patients (60%), and subarachnoid hemorrhage in two patients (4.4%). The average time from symptom onset to SDAVF diagnosis was 18.5 months (range, 11–40 months). The responsible arteries were the heel medullary branches of the medullary arteries, characterized by the thoracic intercostal and lumbar heel arteries, which were observed in 39 patients (86.7%).

**Table 1:** Clinical characteristics of SDAVF patients

Variables	Value
Gender (female, *n*, %)	15(33.3)
Age (years)	52.6 ± 14.3(17 ~ 75)
Hypertension (*n*, %)	14(31.1)
Diabetes (*n*, %)	8(17.7)
Smoking (*n*, %)	13(28.9)
SDAVF location (*n*, %)	
Cervical	4(8.9)
Thoracic	26(57.8)
Lumbar	13(28.9)
Sacrum	2(4.4)
Onset symptom (*n*, %)	
Gait abnormality	41(91.1)
Sensory abnormality	31(68.9)
Urinary dysfunction	27(60.0)
SAH	2(4.4)
Surgical time (h)	4.5 ± 0.6(3.5 ~ 8)
Days of hospitalization (days)	20.5 ± 8.5(11 ~ 35)
mALS improvement rate (*n*, %)	
1–2 days after surgery	16(35.6)
1 week after surgery	12(26.7)
Follow-up	7(15.6)
mALS outcome at follow-up (*n*, %)	
Improved	35(77.8)
Unchanged	8(17.8)
Worsened	2(4.4)

*SAH* Subarachnoid hemorrhage

The operation time ranged from 3.5 h to 8 h, with an average of 4.5 h ± 0.6 h, and no patients died. The hybrid operation achieved the complete removal of all SDAVF cases. The hospital stay ranged from 11 to 35 days, with an average of 20.5 ± 8.5 days. After surgery, the patient’s condition deteriorated in two cases, which were located in the T12 and L2 segments. The patients showed bladder and rectal sphincter dysfunction, loss of sensation, and decreased muscle strength in the lower limbs, which were considered to be caused by spinal cord edema and degeneration. After surgery, the mALS scores improved in 16 cases (35.6%) at 1–2 days, 12 cases (26.7%) at 1 week, and seven cases (15.6%) at the 6-month postoperative follow-up. At the 6-month postoperative follow-up, no recurrence was observed on re-examination of the spinal cord MRA. When compared with the preoperative mALS score, 35 cases (77.8%) showed significant symptomatic improvement, eight cases (17.8%) remained unchanged, and two cases (4.4%) showed deterioration at follow-up (Table 1).

### Comparison of mALS, HAMD, HAMA, mRS, VAS, and Barthel scores before and after surgery

Compared with the preoperative data, the mALS score [postoperative vs. preoperative: 2(1,3) vs. 3(2.4)], HAMD score [(12.2 ± 5.5) vs. (19.6 ± 6.3)], HAMA score [(15.6 ± 5.5) vs. (20.5 ± 6.5)], and VAS score [3(2,5) vs. 5(4,8)] at the 6-month postoperative follow-up were significantly lower, while the Barthel score was significantly higher [(74.6 ± 8.7) vs. (67.8 ± 9.2)] (*P* < 0.05). The mRS score was lower than the preoperative data [1 (1,2) vs. 2 (1,2.5)], but the difference was not statistically significant (*P* > 0.05). When compared with perioperative outcomes, there was a significant increase in the proportion of “good” neurological outcomes at follow-up (62.2% vs. 33.3%) (*P* = 0.023) (Table 2).

**Table 2:** Comparison of preoperative and postoperative mALS, HAMD, HAMA, mRS, VAS, and Barthel scores

Variables	Preoperative	Postoperative	*t*/*T*/*χ*^2^	*P* value
mALS score	3(2,4)	2(1,3)	− 3.690^c^	< 0.002^*^
HAMA score	19.6 ± 6.3	12.2 ± 5.5	5.973^a^	< 0.001^*^
HAMD score	20.5 ± 6.5	15.6 ± 5.5	3.770^a^	< 0.001^*^
VAS score	5(4,8)	3(2,5)	− 4.615^c^	< 0.004^*^
Barthel score	67.8 ± 9.2	74.6 ± 8.7	− 3.616^a^	< 0.001*
mRS score	2(1,2.5)	1(1,2)	− 0.833^c^	0.405
Neurological outcome (*n*, %)			7.533^b^	0.023^*^
Good	15(33.3)	28(62.2)		
Mild to moderate disability	27(60.0)	14(31.1)		
Severe disability	5(11.1)	3(6.7)		

^*^P < 0.0 5

## Discussion

SDAVF is the most common spinal vascular malformation, with an incidence of approximately 60–80%, and is prevalent in middle-aged and elderly individuals. Due to nonspecific clinical features, patients often visit orthopedic clinics, delaying diagnosis and appropriate treatment [16–18]. Gait abnormalities are the most common symptom of SDAVF [19]. In this study, gait abnormalities were present in 91.1% of the cases, followed by abnormal sensation in the lower limbs in 68.9%, and sphincter dysfunction in 60%. To date, the diagnosis of SDAVF mostly relies on spinal angiography, which is relatively difficult to perform clinically. In this study, the average time between symptom onset and final diagnosis was 18.5 months, which is consistent with previous studies [20]. The newly developed whole spinal cord three-dimensional variable flip angle fast spin echo T2-weighted sequence (3D-T2-SPACE) imaging technique has been used for the definitive diagnosis of SDAVF and is expected to promote the rapid diagnosis and treatment of SDAVF [21]. 

The treatment of SDAVF includes microsurgery, interventional therapy, and hybrid surgery. Currently, interventional therapy is a common modality, but it is not suitable for patients with tortuous arterial routes who are prone to incomplete embolism [22]. Consequently, the disadvantages of endovascular therapy, such as treatment failure, the inability to achieve total occlusion, and recurrence, should not be ignored^7^. Microsurgery requires clear localization of the responsible arteries to prevent catastrophic complications, such as injury to the spinal arteries, in case of an inaccurate judgment during surgery [23]. Hybrid surgery accurately localizes and verifies the responsible arteries, drainage veins, and residuals during the surgery, enhancing the accuracy and reliability of SDAVF resection [24]. Furthermore, we speculated that the hybrid surgery reduced the technical difficulty associated with SDAVF lesions with tortuous feeding arteries, which was beneficial for preserving spinal function. In this study, we achieved complete removal of the SDAVF and observed no recurrence in any patient during follow-up. Previous studies have reported an overall cure rate of approximately 50% and an improvement rate of approximately 30% after surgery [1]. However, in this study, the overall “improvement” rate of SDAVF was 77.8%, with 17.8% of cases showing no change, highlighting the positive role of the hybrid surgery platform for SDAVF. After surgery, the percentages of patients with SDAVF who had “improved” spinal function at 1–2 days, 1 week, and 6 months were 35.6%, 26.7%, and 15.6%, respectively, which was considered a favorable result. However, two patients had loss of muscle strength in both lower limbs and bladder sphincter dysfunction due to spinal cord edema, which did not improve significantly after 6 months of neurological rehabilitation. Future research should explore spinal cord functional stimulation or stem cell therapy for treating SDAVF patients with spinal cord dysfunction [25, 26].

Moreover, this study also reported the results of anxiety, depression, quality of life, and neurological outcomes of SDAVF, which were less frequently mentioned in previous studies. When compared with the preoperative results, the mALS score, HAMD score, HAMA score, and VAS score were significantly lower at the 6-month follow-up, while the proportion of “good” neurological outcomes and the Barthel score was significantly higher (*P* < 0.05). Therefore, hybrid surgery contributes to a positive value of pain relief, improvement of spinal cord function, reduction of anxiety and depression, and improvement in independent living status. However, there was no statistically significant difference in the mRS scores compared with the preoperative scores (*P* > 0.05). We hypothesized that the long-term quality of life is related to the severity of spinal cord dysfunction [27]. The therapeutic process is more complex and recovery from spinal cord functional impairment is slower. Consequently, we speculate that the recovery of patients with SDAVFs is a long-term process that requires multidisciplinary interventions, such as clinical, nursing, psychological intervention, and neurorehabilitation.

This study has several limitations. First, the included SDAVF cases were limited, and the number of cases needs to be further expanded in the future. Second, the scoring scales used in this study did not collect data before discharge, which did not reflect the dynamic changes in the spinal function improvement after surgery. Third, this study did not combine imaging examinations such as diffusion tensor imaging (DTI) or diffusion kurtosis imaging (DKI) techniques, which could not interpret the scale score.

## Conclusion

Hybrid surgery is a safe and effective treatment for patients with SAVF. It is conducive to the precise treatment of SDAVF, helping to improve anxiety and depression, relieve pain, and improve spinal cord function and neurological status. However, treating patients with SDAVF is a complex, long-term process that requires multidisciplinary intervention.

## Abbreviations

DSA: digital subtraction angiography
DTI: diffusion tensor imaging
HAMA: Hamilton Anxiety Scale
HAMD: Hamilton Depression Scale
ICG: indocyanine green
mALS: modified Aminoff-Logue score
mRS: modified Rankin Scale
VAS: Visual Analog Scale
